# Multiple localization of granular cell tumour: a case report

**DOI:** 10.4076/1757-1626-2-8751

**Published:** 2009-09-08

**Authors:** Giovanni Francesco Marangi, Vito Toto, Igor Poccia, Pierluigi Gigliofiorito, Beniamo Brunetti, Paolo Persichetti

**Affiliations:** 1 Department of Plastic and Reconstructive Surgery, University Campus Bio-Medico of Rome, Via Alvaro del Portillo, 00128 Rome, Italy

## Abstract

**Introduction:**

Granular cell tumour, also known as Abrikossoff's tumour, is a rare entity occurring in the skin as well as in internal organs, more common among the third to fifth decade of life. It has often been described as a peripheral neuroectodermal tumour and its clinical behaviour is usually benign although malignant and multifocal forms are also known to occur.

**Case presentation:**

We report a case of multiple granular cell tumour in a 17-year-old Caucasian woman who presented with a nodular lesion in the popliteal cave, diagnosed as an Abrikossoff's Tumour, followed 2 years later by another soft tissue mass in the neck.

**Conclusion:**

This case is quite rare: only a few cases were presented in literature with an extended period of time before the clinical presentation of subsequent disease.

## Introduction

Granular cell tumour (GCT) is an uncommon soft tissue neoplasm, first described by Abrikossoff in 1926 [1]. Females are slightly more affected than males and it is common in the third to the fifth decade of life [2,3]. It can also occur in childhood but this is rare. These neoplasms arise in different parts of the body, particularly in the tongue, subcutaneous tissue and breast. The head and neck site most likely to be involved is the larynx. Other sites such as the gastrointestinal, urogenital, and respiratory tracts can also be involved. Granular cell tumours usually present as a benign solitary nodule. Malignant granular cell tumours (MGCTs) are extremely rare, high-grade sarcomas of Schwann cell origin. They often metastasize and are associated with short survival. Clinical manifestations of granular-cell tumours may vary from showing normal, hyperpigmented or ulcerated skin associated with frequent subcutaneous nodules. They can also be either pruritic or painful. Initially, they were thought to arise from skeletal muscle because of the cytologic resemblance to myocytes. Recent studies suggest its derivation from Schwann cells.

We report a rare case of GCT in a young woman which first presented with a nodular lesion in the left popliteal cave and two years later showed a neck subcutaneous nodule.

## Case presentation

**Figure 1:**
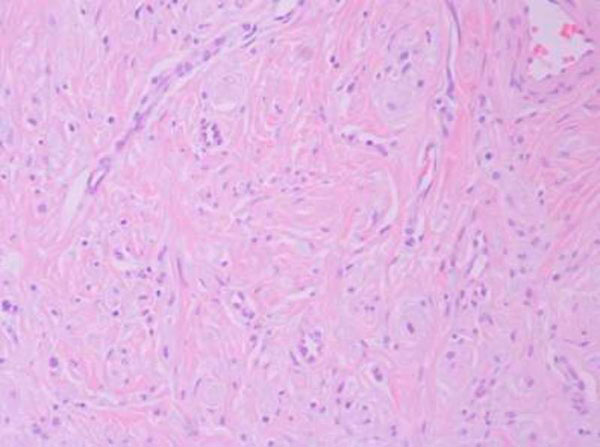
**Histological section stained with hematoxylin & eosin (X 20)**.

**Figure 2:**
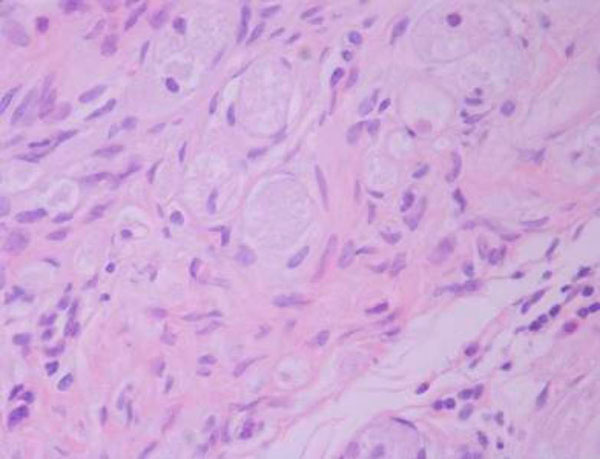
**Histological section stained with hematoxylin & eosin (X 40)**.

A 17-year-old Caucasian woman presented to our department with a palpable lesion in the left popliteal cave. Family history and past medical history were not of significant interest. The lesion appeared 9 months as a 0.5 cm mass, and had slowly enlarged, evolving into a 2 × 1 cm mobile subcutaneous nodule, with clinically distinct margins. The tumour was covered by apparently normal skin. At first the lesion was clinically suspected to be a fibrolipoma and so a simple surgical excision was performed. Histological examination revealed that large polyhedral cells arranged in sheets, with eosinophilic granular cytoplasm. Small oval nuclei were eccentrically placed. Mitotic activity was < 2/10 high power field (HPF). Histological features were suggestive of granular cell tumour. Immunohistochemical study of the nodule was positive for S100 protein, CD68 and p53 in less than 30% of cells. Surgical margins were positive for tumour involvement so a second radical excision including the skin and the subcutaneous tissue was performed. Second histopathological examination assured total excision (Figures 1 and 2).

Approximately 2 years later, a second nodule appeared on her neck. Physical examination revealed a 2 cm subcutaneous, mobile, painless nodule. The lesion gradually enlarged within 6 months. The surrounding skin was normal. Total excision of the nodule was performed. The histopathological examination was identical to the previous specimen showing nests of polygonal cells with abundant eosinophilic granular cytoplasm. There was no cytologic pleomorphism and mitotic activity was >2/10 HPF. The neoplasm was positive for S100 protein and CD68. The patient was discharged on post-operative day 2 with no complication reported. The surgical wound healed in 2 weeks with normal scarring.

In a two years follow up, the patient is in good health without signs of further tumour development.

## Discussion

Granular cell tumours are usually solitary and usually located in the skin or the submucosa of the tongue. The majority of cases involve the head and neck [4,5]. Less frequently involved is the upper respiratory tract [6] and in 10-15% of cases the gastrointestinal tract [7]. Other rare locations include thyroid, breast [8,9], parotid glands, eyes, urinary bladder [10], reproductive organs, abdominal wall [11], cranial and peripheral nerves. It has been also described a correlation with neurofibromatosis, in which multiple subcutaneous nodules are present, especially in children. The neoplasm is typically slow growing, well circumscribed, firm and rounded, with a diameter ranging from 5 to 20 mm, although larger tumours may be seen. The skin surrounding the tumour is usually normal, although it may thicken and rarely may ulcerate. Occasionally tufts of hair may be present on the skin overlying the lesion [12].

The benign tumour consists of large polyhedral cells arranged in sheets with abundant eosinophilic granular cytoplasm. The nuclei are relatively small and mildly pleomorphic with prominent nucleoli. Occasional mitotic figures can be present. The epithelium surrounding the neoplasm often shows pseudoepitheliomatous hyperplasia.

Approximately 1-2 percent of granular-cell tumours are malignant [13,14]. Features suggestive of malignancy include a size greater than 4 cm in diameter, nuclear pleomorphism, lymph nodes metastases, aggressive clinical behaviour, rapid growth, and ulceration. Histopathological diagnosis of malignancy is based on these six criteria: necrosis, spindling, vesicular nuclei with large nucleoli, increased mitotic activity (> 2 mitoses/10 high-power fields at 200x magnification), high nuclear to cytoplasmic (N:C) ratio, and pleomorphism. Neoplasms that meet three or more of these criteria are classified as malignant, those that meet one or two criteria are classified as atypical, and those that display only focal pleomorphism are classified as benign [14].

Due to low mitotic activity (< 2/10 high power field), not significant nuclear to cytoplasmic (N:C) ratio and no pleomorphism detected, we graded both lesions as benign.

Sites of metastases are mainly regional lymphatic nodes, brain tissue, skeleton and lungs.

Initially Abrikosoff suggested that GCT originate from skeletal muscle cells and so referred to them as myoblastomas; this suggestion probably arose from examination of tumours of the tongue in which infiltration between the striated muscle bundles gave the impression of a muscular origin. Recent studies suggest derivation from Schwann cells of the peripheral nerves, and the presence of S100 protein supports this [15]. The KP-1 monoclonal antibody, which recognises the lysosome-associated glycoprotein CD68, reacts positive with schwannomas and granular cell tumours but not with other neuronal neoplasms [16].

## Conclusion

The presentation of the second lesion in our patient makes this case report quite distinctive. Few cases were previously described with a second presentation such temporally far from the primary neoplasm.

Even if 10-15% of granular cell tumours present with satellite nodules, in our case clonality was excluded due to the different topographic involvement occurred.

Crawford and De Bakey [17] reported a patient in whom metastasis occurred 14 years after initial treatment. Mullins and Magner [18] reported a case of a woman who first had a right flank subcutaneous mass, diagnosed as granular cell myoblastoma, and then developing 2 years later a mass in the controlateral parotid gland. Our patient was free from clinical disease for approximately 2 years before the development of the second neoplasm. The histopathological examination of the second lesion was identical to the previous specimens with no features suggestive of malignancy.

The treatment of choice for GCT is wide local excision. In the malignant variety, the treatment should include wide local excision with regional lymph node dissection and radiological evaluation for metastasis. Radiotherapy and chemotherapy are used with variable success, but their effectiveness remains unproven [12].

## Consent

Written informed consent was obtained from the patient for publication of this case report and accompanying images. A copy of the written consent is available for review by the Editor-in-Chief of this journal.

## Competing interests

The authors declare that they have no competing interests.

## Authors' contributions

GFM and PP performed the surgical procedures and contributed to the analysis of the clinical data. BB performed the histological examination of the lesion. VT and PG contributed to the interpretation of the data and to the discussion of the manuscript. VT was a major contributor in writing the manuscript together with IP. All authors read and approved the final manuscript.
